# Quality of Life and Child’s Autism-Specific Difficulties among Malaysian Main Caregivers: A Cross-Sectional Study

**DOI:** 10.3390/ijerph18189861

**Published:** 2021-09-18

**Authors:** Siti Fairus Asahar, Khasnur Abd Malek, Mohamad Rodi Isa

**Affiliations:** 1 Department of Primary Care Medicine, Faculty of Medicine, Universiti Teknologi MARA (UiTM), Sungai Buloh 47000, Malaysia; sitifairusasahar@gmail.com; 2 Department of Public Health Medicine, Faculty of Medicine, Universiti Teknologi MARA (UiTM), Sungai Buloh 47000, Malaysia; rodi@uitm.edu.my

**Keywords:** quality of life, autism spectrum disorders, caregivers, caregiver perceptions, quality of life in autism questionnaire

## Abstract

Caring for children with autism spectrum disorder (ASD) negatively impacts quality of life (QoL). This cross-sectional study aimed to determine the factors associated with perceived QoL and how problematic a child’s autism-specific difficulties are among the main caregivers of children with ASD who attend specialized preschool programs at the National Autism Society of Malaysia and IDEAS Autism Centre located in Selangor and Kuala Lumpur. Utilizing the questions from Parts A and B of the Quality of Life in Autism Questionnaire (QoLA), the data from 116 responders were analyzed using univariate and multivariate linear regression. The mean scores of Part A and Part B were 88.55 ± 17.25 and 56.55 ± 12.35, respectively. The QoL was significantly associated with staying in an apartment/flat −11.37 (95%CI: −19.52, −1.17, *p* = 0.008), main caregivers attending two training sessions 10.35 (95%CI: 1.17, 19.52, *p* = 0.028), and more than three training sessions 13.36 (95%CI: 2.01, 24.70, *p* = 0.022). Main caregiver perceptions of their child’s autistic-specific difficulties were significantly associated with not receiving additional help for childcare: no maid −13.54 (95%CI: −24.17, −12.91, *p* = 0.013); no grandparent −8.65 (95%: −14.33, −2.96, *p* = 0.003); and main caregivers not having asthma 8.44 (95%CI: 0.02, 16.86, *p* = 0.049). These identified factors can be considered to inform main caregivers and health care providers on targeted ways to improve the QoL of main caregivers.

## 1. Introduction

Autism spectrum disorder (ASD) is a lifelong neurodevelopmental disorder characterized by a chronic condition exhibiting deficits in social interaction and communication, restricted repetitive behavioural patterns, interests, and activities in the beginning of childhood [1]. The United States of America observed an increasing prevalence of ASD among 8-year-old children between the years 2000 and 2014, with the prevalence increasing from 6.7 in 1000 children [2] to 16.8 in 1000 children [3]. Prevalence studies on children with ASD within the Asia Pacific region are limited to Japan and China, with an estimated median prevalence value of 11.6 in 10,000 [4]. Due to the limited data availability and the small sample size of these studies, the true prevalence of children with ASD in Asia is not known. Similarly, within the local setting, the lack of an ASD registry limits the knowledge of prevalence. Nevertheless, the number of children with ASD enrolling in early intervention training centres in Malaysia is increasing. The National Autism Society of Malaysia (NASOM), which is the largest local NGO-based autism training centre in Malaysia, reported an increased percentage of children with ASD enrolling in their centre of 30% in 2009 [5].

Behavioural manifestations of ASD commonly appear between the ages of one and three years of age. The symptoms change as the child grows and vary in severity. A child who exhibits communication problems with hyperactivity may develop relationship and mood problems as they grow up into the adolescent phase of life [6]. Furthermore, approximately 70% of the children with ASD have at least one con-commitment psychiatric condition [7]. Commonly reported co-morbidities are psychiatry and maladaptive disorders. The former is related to anxiety disorders and attention deficit hyperactivity (ADHD) [8], while the latter is related to aggression and destructive behaviours [9]. Other associated co-morbidities include poor eating, epilepsy, and gastrointestinal problems [10]. Therefore, parents of children with ASD face long-term multiple challenges due to their child’s developmental difficulties.

The dynamic nature of the challenges faced by caregivers of children with ASD puts them at a greater risk for a negative quality of life (QoL). When the diagnosis of ASD is initially made, caregivers have to learn to accept, adapt, and cope with new information and needs [11]. When the child reaches the ages between four and eight-years-old, caregivers are challenged with new co-morbidities and the child’s increased levels of emotional or behavioural symptoms [12]. Studies worldwide have consistently reported lower QoL levels among parents of children with ASD than parents with typically developing children or even parents with children who have other disabilities [13,14].

Other obstacles faced by the caregivers include the handling their child’s often unpredictable spectrum of behaviour and emotional challenges. Caregivers may find this behaviour problematic to them, and this may lead to caregiver burn-out [15] and chronic stress development [16]. Several factors that impact caregiver QOL have been identified. These include the severity of the core features of ASD, the presence of comorbidities, particularly maladaptive behaviours such as hyperactivity; oppositional defiant and conduct problems; anxiety and emotional symptoms; and the level of general developmental delay and impairment in daily living activities [17].

Many studies have been conducted to assess the QOL of caregivers of children with ASD. However, the subject related to factors associated with QoL among caregivers with children with ASD remains understudied in Malaysia. A conference proceeding reported by Lope [18] found a lower QoL among low-income Malaysian Chinese ethnic parents compared to Malay parents. Another study conducted at a Chinese non-governmental organization (NGO) found that the parent’s gender, employment status, education or income level [19], and intrinsic motivation [20] are associated with higher (QoL). These studies sampled parents who were primarily Chinese in ethnicity, with limited variables being studied. Understanding the QoL and the particular implications of ASD associated symptoms on main caregiver QoL using an ASD specific measure questionnaire is critical in order to provide appropriate support services for parents of ASD children.

In response, our study aims to determine the QoL level, perception of the child’s autism-specific difficulties level, and their associated factors among the main caregivers who send their children to the largest local NGO-based autism training centre. According to the Malaysian National Health System Review [21], a study on QoL is recommended and essential to improving the efficiency of the current services for the community and for children with special needs. Therefore, input from main caregivers about their QoL is crucial in evaluating and improving current ASD services. This study used the Quality of Life in Autism (QoLA) questionnaire to measure the QoL, as it is an ASD-specific questionnaire that measures all of the relevant aspects of living with ASD [22]. This QoLA has two constructs. QoLA Part A measures the overall perceptions that parents have of their quality of life, and QoLA Part B measures parent perceptions of how problematic their child’s autism-specific difficulties are for them.

The following research questions and hypotheses have been put forward:

**Hypothesis 1** **(H1).** *What is the level of QOL among the main caregivers of children with ASD? We expected that the reported quality of life would be lower among the main caregivers of children with ASD.*

**Hypothesis 2** **(H2).** *What are the associated factors for the QoL among the main caregivers of children with ASD? We expected that there would be associated factors for the QoL among the main caregivers of children with ASD.*

## 2. Materials and Methods

### 2.1. Location, Study Design and Sampling

This cross-sectional study collected the data from main caregivers with children with ASD who send their children to the non-governmental organization (NGO) early intervention childcare centres in Selangor and in Kuala Lumpur, Malaysia. Using stratified sampling, one IDEAS Autism Center (IAC) site and five National Autism Society of Malaysia Centre (NASOM) sites were selected. These NGO-led centres offer educational and interventional programs for children with autism to low and medium-income families. Consecutive main caregivers were approached for participation between 1 June and 31 October 2018.

Every main caregiver who attended the NGO early intervention childcare centre was approached in a cubicle room and was invited to partake in the study. Next, they were screened for eligibility according to the inclusion and exclusion criteria. Researchers gave a detailed explanation of the purpose of the study followed by obtaining written informed consent. Following this, consenting eligible participants were given the study questionnaires. A trained research assistant collected the data to guarantee a uniform data gathering procedure. Every submitted questionnaire was checked for completion.

### 2.2. Study Participants and Recruitment

The study included main caregivers whose children were between the ages of three and nine years of age, and the child’s clinical diagnosis of ASD had been confirmed by either a child psychiatrist, paediatrician, or family medicine specialist at least 3 months before the commencement of this study. We excluded main caregivers whose child’s ASD diagnosis was either uncertain or suspected. Researchers approached all main caregivers during the data collection duration and invited those who fulfilled the eligibility criteria to participate in the study. Following this, consenting participants completed a face-to-face administered questionnaire on the sociodemographic and clinical details of the main caregivers (Section 1) and their children with ASD (Section 2) and a self-administered QoLA questionnaire (Section 3).

Section 1 asked about the main caregiver’s age, gender, race, religion, ethnicity, living together with his or her spouse, education level, occupation, medical illness, housing type, number of children, number of children with special needs, frequency of attendance to parent training courses, involvement in parent support groups, medical co-morbidities, and combined household income.

Section 2 asked about the child’s comorbidities related to ASD, additional support in looking after the child, financial aid, frequency of attendance to a specialist clinic, co-morbidities, the accomplishment of urine and bowel training, and for the ability to communicate their needs. In this study, children with ASD were considered to have accomplished urine or bowel training when their main caregivers reported their child’s ability to go to the toilet by themselves and to perform the toileting sequence for urination or motion without direction, assistance, or prompting from others. Toileting sequence is defined as the ability to inform someone of their needs with or without words, finding the toilet, undressing, sitting on the toilet, urination or bowel motion without soiling their pants or underwear, cleaning themselves, flushing the toilet, redressing, washing and drying their hands, and returning to the previous or to a new activity.

Section 3 of the QoLA questionnaire assesses the autism-specific measure of quality of life for parents of children with ASD (Part A) and the parent perceptions of how problematic their child’s autism-specific difficulties symptoms are (Part B). A researcher was available on-site to attend to any query immediately. Participants took about 10 to 15 min to complete the QoLA questionnaire.

### 2.3. Instrument: Quality of Life in Autism (QoLA) Questionnaire

Parents of children with ASD face unique challenges in providing care. The English version on the quality of life in autism (QoLA) questionnaire specific to autism was used in this study. The QoLA questionnaire is an autism-specific measure of QoL for parents of children who are 2–18 years of age. The psychometric properties of QoLA were developed to be sufficiently sensitive to measure the unique challenges faced by parents of children with ASD, particularly concerning social and emotional aspects [22]. The QoLA Part A measured the overall perceptions that the main caregivers had of their quality of life, which consisted of 28 items. QoLA Part B measured parent perceptions of how problematic their child’s autism-specific difficulties symptoms are for them, which had 20 items highlighting the difficulties that children with ASD can experience.

Both parts had strong psychometric properties, with an alpha coefficient of 0.94 for Part A and 0.92 for Part B [23]. For construct validity, it had a significantly lower score in the clinical group compared to in control group for Part A and Part B. The Cohen’s d effect sizes were also big for both parts (1.17 and 2.08, respectively). For concurrent and convergent validity, the scores of QoLA Part 1 were positively correlated with the scores on all four subscales of the WHOQOL-BREF (f = 0.74 to 0.91, all *p* < 0.01). The scores for QoLA Part B approached a significant correlation with the SCQ (r = −0.37, *p* = 0.086).

The final scores for Part A and Part B were calculated by summing the relevant items for both constructs separately, as recommended by the questionnaire developer [22]. Total score for Part A ranged from 28 to 140, with higher scores indicating greater perceived QoL. The total scores for Part B ranged from 20 to 100, with higher scores denoting fewer problems perceived by parents regarding their child’s autistic symptoms [22]. The author of the QoLA questionnaire granted permission for its use in this study.

### 2.4. Operational Definition

In this study, main caregivers were defined as either fathers or mothers who were mainly responsible for caring and providing the needs for their children with ASD and who were the main people responsible for caring for the child and who were actively engaged in providing care and the needs of the child [11].

### 2.5. Sample Size

The sample size was calculated using the single formula method. Dardas [24] used the World Health Organization QoL questionnaire and reported the QoL level among main caregivers with children of ASD in the Arab region to be 61.39 ± 12.57, with 5% absolute precision, power of 80% [25], a standard deviation of 12.57, and an estimated difference from the population mean of 2.5; the required sample size was calculated to be 97. Taking into consideration a non-response rate of 20%, the required sample size was 116.

### 2.6. Statistical Analysis

Data were managed and analyzed using the SPSS Version 24.0. In the descriptive analysis, normally distributed data were presented in mean and standard deviation while non-normally distributed data were presented in median and interquartile range (IQR). Categorical data were presented in frequency (*n*) and percentage (%). The responses for each item of the main caregivers towards the Quality of Life in Autism (QoLA) questionnaire for Part A and Part B were presented in terms of frequency (*n*) and percentage (%). The total scores of Part A and Part B were presented in terms of mean and standard deviation. Univariate analysis was used to determine the factors associated with perceived quality of life (QoLA) among the caregivers, and the factors associated with how problematic the child’s ASD-specific difficulties were analysed using simple linear regression (SLR). Independent variables with less than 0.05 were included for further analysis using multiple linear regression (MLR) to adjust for the confounding factor. The MLR was analyzed using the backward method. All assumptions were tested accordingly, including checking for model fits, the interaction between the independent variables, and multicollinearity. The final model was assessed for overall model fit using linearity, independence samples, normality of the residuals, and homodescasticity. R^2^ was presented to determine how much the dependent variable was explained by the variance of the significant independent variables. The level of statistical significance was set at *p* < 0.05.

### 2.7. Ethical Consideration

Ethical approval was obtained from the Research Ethics Committee, Research Management Institute, UiTM (600-IRMI (5/1/6)). Approvals from the National Autism Society of Malaysia (NASOM) and the IDEAS Autism Centre (IAC) were obtained.

## 3. Results

### 3.1. Sociodemographic and Clinical Characteristics of the Main Caregivers

Out of 126 main caregivers who were approached, 116 completed the questionnaires, giving a response rate of 92.1%. The mean age of the main caregivers was 35.77 ± 3.10 years. The vast majority of the questionnaire was completed by mothers (80.2%). Many were Malay (94.8%) in ethnicity, Muslim (94.8%), and had at least a secondary school education (15.5%). Approximately 92.2% of the main caregivers lived together with a spouse and stayed in an apartment/flat (33.6%). Many worked in the government sector (35.2%), and their total monthly household income was between 3000 MYR to 4999 MYR (28.4%). About three-quarters of the participants were already involved in parent training workshops (79.3%) and support groups (87.1%). Less than half (47.4%) of the main caregivers had extra support for their children with autism. Just over a quarter of them had medical co-morbidities (25.9%), with depression (17.2%) and anxiety (14.7%) being the common conditions reported. Table 1 shows the sociodemographic and clinical characteristics of the main caregivers.

### 3.2. Clinical Characteristics of ASD Children

More than half of the children (53.4%) had at least one co-morbidity. All of children had an eating disorder (100%) followed by ADHD (48.4%). Many children attended regular specialty clinics (74.15%), with speech therapy clinics being the most commonly visited (83.7%) clinics. Just under three-quarters were able to handle their urination needs (68.1%), and half of the children (50.9%) were able to handle their bowel motion needs. The clinical characteristics of ASD children can be found in Table 2.

### 3.3. The Response of the Quality of Life in Autism (QoLA) Part A and Part B

The internal consistency of the QoLA was adequate, with *α* = 0.86 for Part A, and *α* = 0.87 for Part B, which indicated that the questionnaire had good internal consistency in our study. The mean QoL score (Part A) was 88.55 ± 17.25, with a minimum score of 40 and a maximum score of 130. The score of the perceptions that the main caregivers had regarding the difficulties related to their child’s ASD symptoms (Part B) was 56.55 ± 12.35. The lowest score was 23 while the highest score was 82. Table 3 shows the main caregivers’ responses to each item of QoLA Part A and QoLA Part B.

### 3.4. Univariate Linear Regression and Multiple Linear Regression Analysis for Factors Associated with Perceived Quality of Life (QoLA Part A) among the Main Caregivers

Univariate linear regression analysis was conducted to determine the factors significantly related to the perception of QoL among caregivers (Part A). Twelve significant factors with *p* < 0.05 were: race (*p* = 0.009); religion (*p* = 0.009); educational status (*p* = 0.022); type of house (*p* = 0.014); frequency of attending training (*p* = 0.009); support group on Facebook (*p* = 0.036); support group on Whatsapp (*p* = 0.006); child’s siblings helping to look after the child (*p* = 0.005); main caregivers with anxiety (*p* = 0.043); slow learner (*p* = 0.028); follow up at paediatric clinic (*p* = 0.025); and child talks in sentences (*p* = 0.022). These twelve factors were entered into the multivariate linear regression model. The overall regression model had a good fit for the data. The model identified three significant factors associated with QoL Part A. Among the main caregivers, perceived QoL for Part A was lower when the participants indicated that they stayed in an apartment/flat −11.37 (95%CI: 1.17, 19.52, *p* = 0.008) compared to the main caregivers staying in a single-storey house. Perceived QoL was higher when the main caregivers attended more training sessions compared to those who attended training only once: for two training sessions 10.35 (95%CI: 1.17, 19.52, *p* = 0.028); for more than two training sessions 13.36 (95%CI: 2.01, 24.70, *p* = 0.022). These factors were statistically significant in predicting QoLA Part A among the main caregivers F(5, 39) = 3.041, *p* < 0.05, R^2^ = 0.188. A summary of all of the significant factors associated with QoLA Part A is displayed in Table 4.

### 3.5. Univariate Linear Regression and Multiple Linear Regression Analysis for the Perceived Problem of the Child’s ASD Difficulties QoLA (Part B) among the Main Caregivers

Univariate linear regression analysis was conducted to determine the factors that were significantly related to the perceptions among caregivers for how problematic their child’s ASD difficulties were for them (Part B). The eleven significant factors with *p* < 0.05 were race (*p* = 0.031); religion (*p* = 0.031); type of house (*p* = 0.046); frequency attending training (*p* = 0.049); support group on Facebook (*p* = 0.022); maid helps to look after the child (*p* = 0.029); child’s grandparents help to look after the child (p = 0.005); main caregivers with anxiety (*p* = 0.024); main caregiver having asthma (*p* = 0.039); follow up at paediatric clinic (*p* = 0.025); and child talks in sentences (*p* = 0.022). These eleven factors were entered into the multivariate linear regression model. The overall regression model had a good fit for the data. The model identified three significant factors associated with QOL Part B. The main caregivers’ perceptions of problems caused by their child’s autism symptom difficulties were greater in caregivers who had no maid −13.54 (95%CI: −24.17, −12.91, *p* = 0.013) and no grandparents −8.65 (95%CI: −14.33, −2.96, *p* = 0.003) to help care for their child compared to caregivers who had additional help. The main caregiver’s perceptions of problems caused by their child’s autism symptom difficulties were lower in caregivers who had no asthma 8.44 (95%CI: 0.02, 16.86, *p* = 0.049) than they were in those who had asthma. These factors were statistically significant in predicting QoLA Part B among the main caregivers F(5, 86) = 3.041, *p* < 0.05, R^2^ = 0.239. A summary of all of the significant factors associated with QoLA Part B is displayed in Table 5.

## 4. Discussion

In this study, we investigated the level of QoL among the main caregivers of children with ASD attending NGO autism interventional centres in Selangor and Kuala Lumpur, Malaysia using the QoLA autism-specific questionnaire. QoLA Part A and Part B were independently discussed.

The QoLA Part A construct measures the overall pereptions that parents have about their quality of life [22]. The overall score for Part A in this study was 88.55 ± 17.25. The level of QoL was marginally higher than that reported by Eapan [22] at 86.96 ± 21.53 and was slightly lower than that of Due [26] at 91.6 ± 13.7. The differing exposure duration to interventional services could explain the slightly higher level of QoL among our study samples as well as the levels seen in Due [26] compared to that of Eapen [22]. Our study and the Due [26] study recruited main caregivers whose children were already established in a program. In contrast, Eapen [22] recruited women whose children were on a waiting list or who had had just joined the program within a month’s time. The duration of exposure to support services offered to children with ASD has been associated with a lower level of stress among parents [27], which potentially results in a better QoL.

This study found two factors were significantly associated with Part A: staying in an apartment/flat and attending parent training. The main caregivers of children with ASD living in an apartment/flat was negatively associated with QoL when compared to those staying in a single-story house. Similar findings from previous studies using the QoLA questionnaire have not been described. However, our findings support an earlier study investigating the perceived satisfaction of parents of children with intellectual disabilities in Beijing, China, using the Family Quality of Life Scale. Using multivariate analysis, Hu [28] found a significant difference in their post hoc analysis between families living in a small, crowded house setting compared to those living in a large living setting. Other than space, housing conditions with insufficient living space and that were lacking basic standards, for example, have been identified to be associated with increased psychological distress among the mothers of children with ASD in the United Kingdom [29]. In Malaysia, apartment or flat housing is defined as a walk-up four-story housing block or a high-rise housing block equipped with or without lifts [30], suggesting that apartments or flats in Malaysia are more likely to have a constrained outside space with limited facilities. It seems apparent that living space and conditions have a detrimental effect on QoL, especially among parents of children with disabilities, even when housing conditions are defined and measured differently.

Furthermore, our study also showed a significant positive association between attending two or more parent training sessions with QoL compared to those attending just one training session in a year. While there is no direct comparison to other previous studies using QoLA, a systematic review analyzing parental QoL suggested that interventions from which parents of children with ASD can benefit from to break the child’s challenging behavioural cycle can potentially improve psychological outcomes for the whole family [31]. In a study by Keen [32], a brief intervention for parents of children with ASD aged 2–4 years whose diagnosis was made within six months consisting of two workshops and twice-weekly home visits showed a significant effect on reducing child-related parenting stress. These findings indicated that activity targeting at handling stress as well as difficult ASD difficulty symptoms and behaviour may be able to reduce child-related parenting stress, which may, in turn, improve QoL.

The QoLA Part B construct measures the main caregivers’ perceptions of how problematic their child’s autism-specific difficulties are for them [22]. The overall score of QoLA Part B was 56.55 ± 12.35, which was comparatively lower than the studies conducted among parents whose ASD children were attending government-led autism centres in Tasmania/Adelaide and Sydney at 58.7 ± 16.0 [26] and 68.52 ± 17.56 [22], respectively. The lower score in this study may indicate that the main caregivers in our study may have perceived their child’s autistic symptoms to be more of a problem to them. The two most common items that the main caregivers in this study found to be very much a problem were item B1 “Child socialising with people” and item B12 “Child understands the rules of social interaction”. Since QoLA Part B is rather unique, in that it asks the parents of children with ASD on how much a problem in a range of ASD-specific behaviours is for the parent, identifying studies that compared this construct directlywas not possible. Nevertheless, previous studies found that parents of children with ASD reported a higher burden load compared to the parents of a child with other disabilities and medical conditions such as Down’s Syndrome and Type 1 Diabetes Mellitus. Specifically, impaired adaptive functioning among children with ASD created problems in initiating and maintaining social bonding with similar-aged children, which added to a burden due to a loss of social support [33].

Identifying studies using a psychometric measure similar to QoLA Part B was very challenging. The comparison made for the findings of this study used a reference that measured how much a problem in a range of ASD-specific behaviours is for the caregiver. This study identified three significant factors associated with QoLA Part B, which were “no maid to look after the child”, “child’s grandparent not helping to look after the child”, and “main caregivers without asthma”. Main caregivers not receiving help in looking after their child with ASD was negatively associated with the perception of problems with their child’s difficulties related to their autism symptoms when compared to the main caregivers who had support looking after their children. This finding is comparable to a study conducted among the primary caregivers of children with ASD in the United States, which involved children who were between the ages of 4- to 17-years-old. The study used a care-related quality of life instrument to measure the impact of caregiving and found that a higher impact of caring for children with ASD was positively associated with higher subjective burden and lower family quality of life [34]. Parents of children with ASD faced continuous, daily caregiving demands in delivering complex care needs, leading to a high level of stress and great emotional pressure. The inability to have caregiving support may escalate stress levels and may pose family adjustment difficulties, leading to the inability to cope and the risk of crisis development [35]. Receiving less support in caring for their children is of concern, as it can negatively impact the caregiver and can lead to anxiety and depression [36]. In contrast, having additional caregivers at home can help reduce the caregiving burden [37], and can provide positive fulfilment towards completing caregiving tasks [38].

Concerning the presence of chronic health conditions, we found that main caregivers without asthma were positively associated with improved perceptions of their child’s autism-specific difficulties. Caregivers of a child with ASD shoulder a constant and complex caregiving routine, which could lead to feeling stressed and burdened [39]. A population-based study on caregiver quality of life found that caregivers with and without the presence of chronic diseases suffer from negative physical and mood impairment [40]. However, caregivers who are physically fit and healthy are more likely to have a better QoL, life satisfaction, and well-being [41]. Therefore, well and healthy caregivers are important factors for caregivers, which may help them better handle the stress and the roller coaster of conflicting emotions that come with caring for their children with ASD.

As far as the researchers are concerned, this was the first local study conducted in the largest NGO-based autism training centre in the Selangor and Kuala Lumpur states in Malaysia that studied a subject related to QoL among the main caregivers of children with ASD. It adds to the current body of knowledge by investigating multiple independent variables associated with the QoL of main caregivers, which had not been previously studied within the local setting. However, this study has a few limitations. The cross-sectional study design precluded the analysis of cause–effect relationships. Secondly, convenient sampling may predispose this study to selection bias. However, measures were taken to reduce the sampling bias by ensuring that every main caregiver in the NGO-based autism training centre registry was approached for participation during the data collection days. The representativeness of the sample in this study was limited, as the data contained a large proportion of mothers as the main caregivers, and the study setting was concentrated in urban areas, which may carry a unique social, ethnic, and economic status. Thus, data from this study cannot be generalized to the entire population of Malaysia, but it can explain the needs of Malay mothers as the main caregivers of child with ASD in the urban areas who share the same culture and ethnicity profiles. Furthermore, the study only focused on families of young children with ASD and did not represent the perception of those families with older children with ASD. The lack of significance for some of the independent variables in this study may be due to methodological challenges and limitations in extracting information via a self-administered questionnaire. The findings in this study were based on caregiver reports. As such, inaccurate reporting might have biased our findings. The R^2^ of 18.8% and 23.9% indicate that there were other influencing independent variables towards QoLA Part A and QoLA Part B, respectively, that were not investigated in this study. Given that human behaviour varies and cannot be accurately predicted, this study provides a useful clinical model for data trends among this sample population but may be low to moderate in terms of precision. Finally, we found that there are emerging data from other studies using the QoLA questionnaire, but published studies with similar independent variables to our study are limited, which affects the overall scope of discussion in our study. The complexity of the factors that are relevant to the QoL of main caregivers of children with ASD within the local context is an area of opportunity for further research.

## 5. Conclusions

Based on the information obtained in this study, several factors that positively or negatively affect the QoL of caregivers of patients with autism and their perception of how problematic their child’s autism-specific difficulties symptoms are were determined. Among these factors, those for which an association was established were staying in an apartment/flat, attending two or more training sessions, receiving help to care for the child with ASD, and not having asthma. These factors can be used by main caregivers and health care providers to develop strategies to reduce the burden of caring for children with ASD.

## Figures and Tables

**Table 1:** Sociodemographic and clinical characteristics of the main caregivers (*n* = 116).

Variables	Frequency, *n* (%)	Mean ± SD
Age, years	-	35.77 ± 3.10
Gender		
Male	23 (19.8)	-
Female	93 (80.2)	
Race		
Malay	110 (94.8)	-
Non-Malay	6 (5.2)	
Religion		
Muslim	110 (94.8)	-
Non-Muslim	6 (5.2)	
Spouse		
Having a spouse	107 (92.2)	-
Not having a spouse	9 (7.8)	
Educational status		
Secondary school	18 (15.5)	-
Certificate/diploma	30 (25.9)	
Degree	51 (44.4)	
Postgraduate	17 (14.7)	
Occupation		
Not working/housewife	33 (28.4)	-
Own business	14 (12.1)	
Private sector	27 (23.3)	
Government sector employee	42 (35.2)	
Household income		
MYR 1000–MYR 1999	11 (9.5)	-
MYR 2000–MYR 2999	17 (14.7)	
MYR 3000–MYR 4999	33 (28.4)	
MYR 5000–MYR 6999	23 (19.8)	
MYR 7000–MYR 9999	23 (19.8)	
More than MYR 10,000	9 (7.8)	
Type of house		
Single-storey	33 (28.4)	-
Double-storey	38 (32.8)	
Apartment/flat	39 (33.6)	
Condominium	6 (5.2)	
Total number of children		
1	32 (27.6)	-
2	39 (33.6)	
3	32 (27.6)	
More than 4	13 (11.2)	
Number of special need child		
1 child	111 (95.7)	-
2 children	5 (4.3)	
Attending parent training workshop		
Yes	92 (79.3)	-
No	24 (20.7)	
Frequency attending training		
1	42 (45.7)	-
2	33 (35.9)	
3 and above	17 (18.3)	
Involved in a parent support group		
Yes	101 (87.1)	-
No	15 (12.9)	
Source of support group		
Facebook	96 (82.8)	-
Whatsapp	60 (51.7)	
Others	1 (0.9)	
Support to help look after the child		
Yes	55 (47.4)	-
No	61 (52.6)	
Support to help look after the child		
Child’s grandparent	27 (23.3)	-
Child’s sibling	26 (22.4)	
Child’s auntie	25 (21.6)	
Babysitter	9 (7.8)	
Maid	8 (6.9)	
The child received any allowance		
Yes	63 (54.3)	-
No	53 (45.7)	
Source of allowance (*n* = 63)		
Social welfare	61 (96.8)	-
State government	2 (3.2)	
The main caregiver has medical illness		
Yes	30 (25.9)	-
No	86 (74.1)	
Main caregivers with co-morbidities		
Depression	20 (17.2)	-
Anxiety	17 (14.7)	
Hypertension	11 (9.5)	
Asthma	9 (7.8)	
Others medical illness	4 (3.4)	
Diabetes mellitus	3 (2.6)	
Cholesterol	3 (2.6)	
Heart problem	1 (0.9)	

**Table 2:** Characteristics of the children with ASD (*n* = 116).

Variables	Frequency, *n* (%)
Child of autism has co-morbidities	
Yes	62 (53.4)
No	54 (46.6)
Child of autism with co-morbidities	
ADHD	30 (48.4)
Epilepsy	17 (27.4)
Slow learner	27 (43.5)
Eating disorder	62 (100.0)
Sleep problem	10 (16.1)
Digestive problem	12 (19.4)
ADHD	30 (48.4)
Psychiatric problem	0 (0.0)
Child of autism attending follow-up	
Yes	86 (74.1)
No	30 (25.9)
Distribution attending follow-up	
Speech therapy	72 (83.7)
Occupational therapy	70 (81.4)
Paediatric clinic	54 (62.8)
Child psychiatric clinic	45 (52.3)
Dental clinic	17 (19.8)
ENT clinic	12 (14.0)
Physiotherapy	4 (4.7)
Handling urine her / himself	
No	17 (14.7)
With help	20 (17.2)
Yes	79 (68.1)
Handling bowel motion her/himself	
No	30 (25.9)
With help	27 (23.3)
Yes	59 (50.9)
The measure used to communicate	
Talk in word or sentences	71 (61.2)
Using gesture	71 (60.3)
Picture exchange communication (PECS)	31 (26.7)
Use sign language	8 (6.9)

**Table 3:** Responses of the main caregivers to the Quality of Life in Autism (QoLA) questionnaire for Part A and Part B (*n* = 116).

**QoLA Part A Items**	**Scale** **(Very Much of a Problem for Me) (Not Much of a Problem for Me)**				
	**1**	**2**	**3**	**4**	**5**
	***n* (%)**	***n* (%)**	***n* (%)**	***n* (%)**	***n* (%)**
A1 Satisfied with life	2 (1.7)	16 (13.8)	35 (30.2)	45 (38.8)	18 (15.5)
A2 Feel stressed *	9 (7.8)	15 (12.9)	47 (40.5)	34 (29.3)	11 (9.5)
A3 Feel happy and content	6 (5.2)	12 (10.3)	41 (35.3)	41 (35.3)	16 (13.8)
A4 Feel depressed or anxious *	17 (14.7)	19 (16.4)	45 (38.8)	23 (19.8)	12 (10.3)
A5 Feel good about self or person	17 (14.7)	10 (8.6)	34 (29.3)	38 (32.8)	17 (14.7)
A6 Satisfied with close relationship	6 (5.2)	9 (7.8)	35 (30.2)	42 (36.2)	24 (20.7)
A7 People are there for me when I need	4 (3.4)	16 (13.8)	30 (25.9)	35 (30.2)	31 (26.7)
A8 Satisfied with social life	7 (6.0)	17 (14.7)	33 (28.4)	42 (36.2)	17 (14.7)
A9 Satisfied with family	4 (3.4)	9 (7.8)	28 (24.1)	48 (41.4)	27 (23.3)
A10 Satisfied with financial situation	18 (15.5)	19 (16.4)	45 (38.8)	25 (21.6)	9 (7.8)
A11 Satisfied with where live	3 (2.6)	22 (19.0)	26 (22.4)	45 (38.8)	20 (17.2)
A12 Enough money to meet needs	21 (18.1)	28 (24.1)	35 (30.2)	25 (21.6)	7 (6.0)
A13 Satisfied with achievements	6 (5.2)	19 (16.4)	47 (40.5)	33 (28.4)	11 (9.5)
A14 Satisfied with general health	3 (2.6)	14 (12.1)	45 (38.8)	41 (35.3)	13 (11.2)
A15 Have a healthy lifestyle	9 (7.8)	13 (11.2)	52 (44.8)	30 (25.9)	12 (10.3)
A16 Satisfied with leisure activities	13 (11.2)	21 (18.1)	46 (39.7)	28 (24.1)	8 (6.9)
A17 Health problem stops them do things that they want to *	5 (4.3)	14 (12.1)	31 (26.7)	26 (22.4)	40 (34.5)
A18 Feel in control of life	10 (8.6)	18 (15.5)	44 (37.9)	31 (26.7)	13 (11.2)
A19 Set and achieve goals in life	10 (8.6)	22 (19.0)	41 (35.3)	34 (29.3)	9 (7.8)
A20 Make a plan of action and follow it	4 (3.4)	16 (13.8)	48 (41.4)	37 (31.9)	11 (9.5)
A21 Make own decision	6 (5.2)	13 (11.2)	46 (39.7)	39 (33.6)	12 (10.3)
A22 Feel guilty *	8 (6.9)	12 (10.3)	36 (31.0)	32 (27.6)	28 (24.1)
A23 Part of a community	7 (6.0)	18 (15.5)	43 (37.1)	34 (29.3)	14 (12.1)
A24 Can get the support they need from the community	4 (3.4)	23 (19.8)	53 (45.7)	25 (21.6)	11 (9.5)
A25 Able to get to where they need to	13 (11.2)	24 (20.7)	41 (35.3)	22 (19.0)	16 (13.8)
A26 Feel safe in everyday life	7 (6.0)	15 (12.9)	35 (30.2)	46 (39.7)	13 (11.2)
A27 Feel respected in everyday life	0 (0.0)	16 (13.8)	49 (42.2)	34 (29.3)	17 (14.7)
A28 Satisfied with the availability of health services	12 (10.3)	14 (12.1)	43 (37.1)	32 (27.6)	15 (12.9)
**QoLA Part B Items**	**Scale** **(Very Much of a Problem for Me) (Not Much of a Problem for me)**				
	**1**	**2**	**3**	**4**	**5**
	***n* (%)**	***n* (%)**	***n* (%)**	***n* (%)**	***n* (%)**
B1 Child socialising with people	19 (16.4)	37 (31.9)	36 (31.0)	19 (16.4)	5 (4.3)
B2 Child having friends	23 (19.8)	29 (25.0)	40 (34.5)	21 (18.1)	3 (2.6)
B3 Child understand other’s feelings	24 (20.7)	28 (24.1)	35 (30.2)	25 (21.6)	4 (3.4)
B4 Child holding a conversation	28 (24.1)	32 (27.6)	29 (25.0)	20 (17.2)	7 (6.0)
B5 Child communicating needs	12 (10.3)	23 (19.8)	36 (31.0)	30 (25.9)	15 (12.9)
B6 Child taking a literal meaning of comments	30 (25.9)	34 (29.3)	29 (25.0)	15 (12.9)	8 (6.9)
B7 Child saying things that are socially embarrassing	13 (11.2)	19 (16.4)	26 (22.4)	24 (20.7)	34 (29.3)
B8 Child needs to stick to a routine	17 (14.7)	20 (17.2)	39 (33.6)	28 (24.1)	12 (10.3)
B9 Child being overly interested in a particular topic	16 (13.8)	19 (16.4)	36 (31.0)	37 (31.9)	8 (6.9)
B10 Child getting anxious in a specific situation or during changes	18 (15.5)	20 (17.2)	48 (41.4)	25 (21.6)	5 (4.3)
B11 Child is sensitive to certain sensations	16 (13.8)	27 (23.3)	40 (34.5)	23 (19.8)	10 (8.6)
B12 Child understands the rules of social interaction	26 (22.4)	36 (31.0)	35 (30.2)	14 (12.1)	5 (4.3)
B13 Child is able to manage emotional response	20 (17.2)	30 (25.9)	42 (36.2)	20 (17.2)	4 (3.4)
B14 Child needs to do things a certain way	13 (11.2)	19 (16.4)	54 (46.6)	23 (19.8)	7 (6.0)
B15 Child has destructive behaviour including anger and aggression	13 (11.2)	22 (19.0)	41 (35.3)	28 (24.1)	12 (10.3)
B16 Child showing inappropriate emotional reactions	16 (13.8)	23 (19.8)	51 (44.0)	18 (15.5)	8 (6.9)
B17 Child has unusual repetitive behaviours or body movement	16 (13.8)	20 (17.2)	44 (37.9)	25 (21.6)	11 (9.5)
B18 Child engaging in reckless or tactless behaviour	19 (16.4)	17 (14.7)	42 (36.2)	28 (24.1)	10 (8.6)
B19 Child doing daily living tasks independently	15 (12.9)	22 (19.0)	22 (19.0)	22 (19.0)	13 (11.2)
B20 Child responding when approached socially	14 (12.1)	29 (25.0)	44 (37.9)	21 (18.1)	8 (6.9)

* Item is reverse coded.

**Table 4:** Factors associated with perceived quality of life (QoLA Part A) among main caregivers (*N* = 116).

Variables	Simple Linear Regression ^a^			Multiple Linear Regression ^b^		
	B ^c^ (95% CI)	*t*	*p*-Value	Adj. B ^d^ (95% CI)	*t*	*p*-Value
Race						
Malay	ref		1	-	-	-
Non-Malay	−18.69 (−32.65, −4.72)	−2.650	0.009 *			
Religion						
Muslim	Ref		1	-	-	-
Non-Muslim	−18.69 (−32.65, −4.72)	−2.650	0.009 *			
Educational status						
Secondary school	ref		1	-	-	-
Certificate/ diploma	4.87 (−5.21, 14.93)	0.955	0.341			
Degree	7.34 (−1.92, 16.60)	1.571	0.119			
Postgraduate	13.36 (1.94, 24.78)	2.317	0.022 *			
Type of house						
Single storey	ref		1	ref		1
Double storey	−1.11 (−8.94, 6.71)	−0.282	0.778	2.61 (−11.30, 6.08)	−0.598	0.552
Apartment/flat	−9.82 (−17.60, −2.04)	−2.502	0.014 *	−11.37 (−19.52, −1.17)	−2.812	0.008 *
Condominium	10.67 (−3.93, 25.26)	1.448	0.150	5.77 (−10.31, 21.85)	0.714	0.477
Frequency attending training						
1	ref		1	ref		1
2	8.37 (0.84, 15.89)	2.208	0.030 *	10.35 (1.17, 19.52)	2.281	0.028 *
3 and above	12.47 (3.16, 21.77)	2.663	0.009 *	13.36 (2.01, 24.70)	2.382	0.022 *
Support Group Facebook						
Yes	Ref		1	-	-	-
No	−8.88 (−17.16, −0.61)	−2.127	0.036 *			
Support Group WhatsApp						
Yes	ref		1	-	-	-
No	−8.70 (−14.86, −2.53)	−2.791	0.006 *			
Child’s siblings help to look after the child						
Yes	ref		1	-	-	-
No	10.59 (3.20, 17.98)	2.841	0.005 *			
Main caregivers with anxiety						
Yes	ref		1	-	-	-
No	−9.12 (−17.97 0.27)	−2.042	0.043 *			
Slow learner						
Yes	ref		1	-	-	-
No	−8.90 (−16.81, 0.99)	−2.251	0.028 *			
Paediatric clinic						
Yes	ref		1	-	-	-
No	7.18 (0.93, 13.43)	2.276	0.025 *			
Child talks in a sentence						
Yes	ref		1	-	-	-
No	7.47 (1.08, 13.86)	2.316	0.022 *			

^a^ Simple linear regression. ^b^ Multiple linear regression (R^2^ = 0.188; the model fits well; model assumptions are met; there is no interaction between independent variables and no multicollinearity problem. ^c^ Crude regression coefficient. ^d^ Adjusted regression coefficient; CI = confidence interval. * Statistically significant at *α* = 0.05.

**Table 5:** Factors Associated with how problematic the child’s ASD difficulties (QoLA Part B) are among main caregivers (N = 116).

Variables	Simple Linear Regression ^a^			Multiple Linear Regression ^b^		
	B ^c^ (95%CI)	*t*	*p*-Value	Adj. B ^d^ (95%CI)	*t*	*p*-Value
Race						
Malay	ref		1	-	-	-
Non-Malay	−11.15 (−21.24, −1.05)	−2.187	0.031 *			
Religion						
Muslim	Ref		1	-	-	-
Non-Muslim	−18.69 (−32.65, −4.72)	−2.187	0.031 *			
Type of house						
Single storey	ref		1			
Double storey	−2.02 (−7.81, 3.77)	−0.690	0.491			
Apartment/flat	−5.87 (−11.63, −0.12)	−2.021	0.046 *			
Condominium	−2.50 (−13.30, 8.30)	−0.459	0.647			
Frequency attending training						
1	ref		1			
2	3.50 (−2.24, 9.24)	1.211	0.229			
3 and above	7.11 (0.02, 14.20)	1.992	0.049 *			
Support Group Facebook						
Yes	Ref		1	-	-	-
No	6.91 (1.01. 12.82)	2.319	0.022 *			
Maid helps to look after the child:						
Yes	ref		1	ref		1
No	−9.86 (−18.68, −1.04)	2.215	0.029 *	−13.54 (−24.17, −12.91)	−2.534	0.013 *
Child’s grandparent helps to look after the child:						
Yes	ref		1	ref		1
No	−7.56 (−12.78, −2.34)	2.872	0.005 *	−8.65 (−14.33, −2.96)	−3.025	0.003 *
Main caregivers with anxiety						
Yes	ref		1	-	-	-
No	−7.28 (−13.59, −0.97)	−2.286	0.024 *			
Main caregivers with asthma						
Yes	ref		1	ref		1
No	8.81 (0.44, 17.18)	−2.084	0.039 *	8.44 (0.02, 16.86)	1.993	0.049 *
Paediatric clinic						
Yes	ref		1	-	-	-
No	5.45 (0.99, 9.91)	2.419	0.017 *			
Child talks in a sentence						
Yes	ref		1	-	-	-
No	5.18 (0.59, 9.76)	2.237	0.027 *			

^a^ Simple linear regression. ^b^ Multiple linear regression (R^2^ = 0.239; the model fits well; model assumptions are met; there is no interaction between independent variables and no multicollinearity problem. ^c^ Crude regression coefficient; ^d^ Adjusted regression coefficient; CI = confidence interval. * Statistically significant at *α* = 0.05.

## Data Availability

Data from the study are available upon request.
